# Maladie de Kaposi compliquant une aplasie médullaire

**DOI:** 10.11604/pamj.2014.18.169.1351

**Published:** 2014-06-19

**Authors:** Mouna Lamchahab, Bouchra Oukkache, Sofia Marouan, Asmae Quessar, Said Benchekroun

**Affiliations:** 1 Service d'Hématologie et d'Oncologie Pédiatrique, hôpital 20 Août 1953, CHU Ibn Rochd Casablanca, Morocco; 2 Laboratoire d'Hématologie, CHU Ibn Rochd Casablanca, Morocco; 3 Service Central d'Anatomie Pathologique, CHU Ibn Rochd, Casablanca, Morocco

**Keywords:** Aplasie médullaire, immunosuppresseur, maladie de Kaposi, iatrogène, HHV8, aplastic anemia, immunosuppressor, Kaposi disease, iatrogenic, HHV8

## Abstract

Nous rapportons le cas d'un patient marocain de 40 ans, suivi pour aplasie médullaire sous traitement immunosuppresseur et ayant présenté 6 mois après le début du traitement une MK iatrogène. L'histologie cutanée a permis de poser le diagnostic et était en faveur d'une MK. Le traitement consistait à des cures de Bléomycine avec une évolution favorable. Notre cas illustre l'intérêt d'un suivi régulier des patients sous immunosuppresseurs. Cette forme particulière de MK constitue un vrai défi thérapeutique pour le praticien.

## Introduction

La maladie de Kaposi (MK) anciennement appelé « sarcome de Kaposi » décrite à la fin du XIXème siècle par un dermatologue viennois, Mortiz Kaposi Kohn ayant été considérée comme rare à cette époque et actuellement en nette augmentation depuis la pandémie du VIH [1]. La MK est un processus tumoral angiogénique prolifératif et multifocale à double composante vasculaire et à cellules fusiformes, d'expression cutanée et viscérale. Il s'agit d'une affection virale dont l'agent incriminé étant le HHV8 [2]. On décrit 4 formes cliniques, la forme classique dite méditerranéenne, africaine, liée au virus de l'immunodéficience humaine (VIH) et iatrogénique en rapport avec une immunodépression due à différentes affections ou à leur traitement: transplantation d'organe, maladies de système, hémopathie maligne, néoplasi [1–3]. Dans ce cas, le traitement repose essentiellement sur l'arrêt ou la réduction de l'immunosuppression avec en contre partie un risque accru de rechute de la pathologie sous jacente [4]. Nous rapportons un cas de MK secondaire au traitement immunosuppresseur chez un patient atteint d'aplasie médullaire.

## Patient et observation

**Figure 1:**
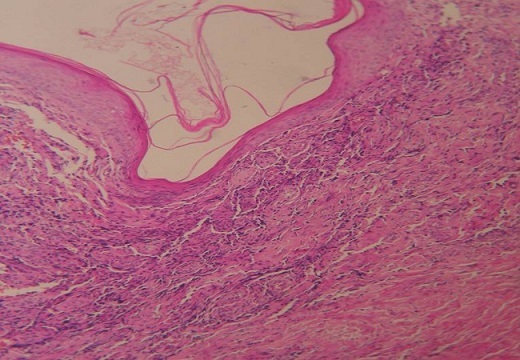
Histologie cutanée montrant une prolifération fusocellulaire, délimitant des fentes et disséquant le derme confirmant le diagnostic de MK (HE x 40)

**Figure 2:**
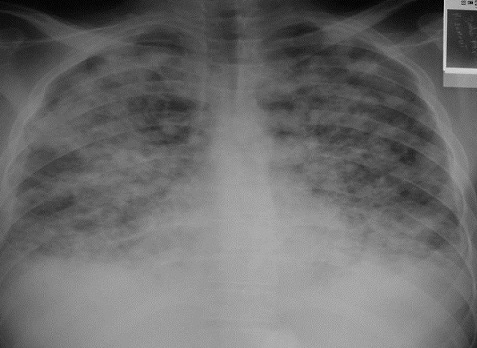
Radiographie du thorax du patient: opacités macro-nodulaires diffuses au niveau des deux champs pulmonaires

**Figure 3:**
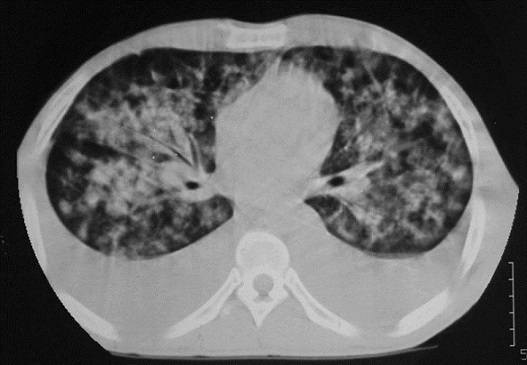
TDM thoracique: syndrome alvéolaire diffus en ailes de papillon compatible avec un œdème lésionnel organisé

Mr Z.M âgé de 40 ans, sans antécédents pathologiques particuliers, a présenté deux mois avant la consultation, un syndrome d'insuffisance médullaire complet. Le bilan biologique objectivait une pancytopénie avec une anémie normochrome normocytaire à 9.6g/dl arégénérative avec un taux de réticulocytes à 20000/ mm^3^, une leucopénie à 700/mm^3^, une lymphopénie à 546/mm^3^ et une thrombopénie à 3000/mm^3^. La biopsie médullaire mettait en évidence une moelle désertique adipeuse concluant à une aplasie médullaire. Le bilan étiologique était négatif concluant à une aplasie médullaire sévère idiopathique. Les sérologies VIH, hépatite B et C étaient négatives. Le patient était traité par Sérum antilymphocytaire (SAL) pendant 5 jours avec ciclosporine à la dose de 6mg/kg/j. Six mois après le début du SAL, le patient avait présenté une dyspnée avec une toux sèche évoluant dans un contexte d'apyrexie avec l'apparition de lésions cutanées diffuses. L'examen clinique objectivait des lésions angiomateuses violacées au niveau des membres évocatrices de MK. L'histologie cutanée était en faveur d'une MK avec une prolifération vasculaire faite de cellules fusiformes localisées. Au niveau dermique, se trouvaient des vaisseaux irréguliers de petites tailles bordées d'un endothélium. Un infiltrat inflammatoire lympho-plasmocytaire périvasculaire avec un dépôt d'hémosidérine était associé (Fig 1). L'immunomarquage au niveau de la peau par les anticorps anti-HHV8 montrait un marquage nucléaire franc et intense de plus de 70% des cellules kaposiennes. La sérologie HHV8 au niveau du sang n'avait pas été réalisée. La radiographie pulmonaire montrait des opacités macro-nodulaires diffuses au niveau des deux champs pulmonaires (Fig 2). Le scanner thoracique objectivait un syndrome alvéolaire diffus en ailes de papillon compatible avec un oedème lésionnel organisé (Fig 3). L’échographie abdominale ainsi que la fibroscopie oeso-gatro-duodénale n'avaient pas retrouvé d'autres localisations viscérales.

Les lésions cutanées s’étaient rapidement étendues avec une aggravation de la gêne respiratoire et des images pulmonaires. La ciclosporine fut arrêtée et des cures de Bléomycine à 15 mg toutes les deux semaines avaient été entreprises. L’évolution était marquée par une bonne réponse clinique avec la disparition totale des lésions cutanées et pulmonaires après 10 cures de Bléomycine. Le patient est en rémission complète de son aplasie médullaire sans séquelles associées avec un recul de 84 mois.

## Discussion

La MK iatrogène a été décrite initialement chez des sujets recevant de fortes doses d'immunosuppresseurs comme les transplantés rénaux [4]. Elle peut également compliquer un simple traitement par corticoïdes ou immunosuppresseurs à faible dose. Le risque de survenue de MK iatrogène chez les patients sous traitements immunosuppresseurs en dehors du contexte de la transplantation est plus faible comparativement avec les patients transplantés [5].

La pathogénie de la MK iatrogène est multifactorielle. Une prédisposition génétique influencerait la réponse immune, qui, stimulée par un agent infectieux, entraînerait une prolifération endothéliale. L′immunosuppression joue un rôle certain, comme l'atteste la survenue d′une MK chez des sujets immunodéprimés à la suite d′une transplantation d′organe, d′une allogreffe de moelle ou de l′infection par le VIH, et le caractère parfois régressif des lésions lorsque l′immunosuppression diminue [6]. L'implication de l'HHV8 dans cette pathogénie pourrait être expliquée par la présence de la protéine Tat, qui exercerait une action directe sur ce virus [7]. Dans notre cas, l'aplasie médullaire ainsi que le traitement par du sérum antilymphocytaire et ciclosporine, représentent les principaux éléments inducteurs dans le développement de la MK.

Le HHV8 été retrouvé dans toutes les variétés de MK, y compris la forme iatrogénique. Zong a pu définir 3 sous types de HHV8: A, B et C. Il existe une épidémiologie particulière à chaque sous type. Au Maroc, le sous type C est le plus fréquent [8]. Chez notre patient, la recherche de l'HHV8 était positive au niveau cutané.

L'origine ethnique des patients atteints de MK iatrogène peut être considérée comme la même que dans la forme classique. La prévalence du MK iatrogène est plus élevée dans les pays méditerranéens et en Afrique. Elle touche préférentiellement les sujets adultes de sexe masculin comme dans la forme méditerranéenne. Selon une étude américaine, cette prédominance masculine pourrait être expliquée par le rôle protecteur du bêta HCG, qui entraînerait une apoptose des cellules des lignées de MK [9].

L'apparition des lésions et leur sévérité sont variables selon le type d'immunosuppresseur utilisé, la posologie et la durée du traitement. Pour notre patient, le délai d'apparition de la MK était de 6mois. Sur le plan clinique, les manifestations cutanées dominent le tableau et ont le même aspect clinique que la forme classique avec une atteinte plus diffuse et plus évolutive touchant essentiellement les extrémités. L'atteinte des muqueuses est fréquente principalement au niveau de la muqueuse buccale décrivant une nappe rougeâtre du palais souvent extensive [10]. Pour notre patient, les lésions étaient principalement de type nodulaire angiomateuse et touchaient principalement les membres. Il ne présentait pas d'atteinte muqueuse.

Les manifestations extra cutanées sont plus fréquentes dans cette forme iatrogénique. Pour cela, et à la recherche d'atteinte extra cutanée, des examens biologiques courants (hémogramme, fonction rénale et bilan hépatique), une radiographie pulmonaire et une échographie abdominale devraient être réalisés systématiquement chez tous les patients atteints de MK iatrogène. Une endoscopie digestive est indiquée en cas d'atteinte de la muqueuse buccale ou en cas d'atteinte étendue ou de siège atypique [11], comme le cas de notre patient, qui présentait une atteinte cutanée généralisée, prédominant au niveau des membres inférieurs, associée à une atteinte pulmonaire.

Le diagnostic de MK est confirmé histologiquement, comme le cas de notre patient. L'aspect histologique associe une prolifération vasculaire tapissée de cellules endothéliales entourées de faisceaux enchevêtrés de cellules fusiformes, et un infiltrat inflammatoire lymphoplasmocytaire, en proportions variables selon le stade de la maladie [10, 11].

Le traitement de la MK iatrogène est sujet de discussions, puisqu'il consiste en une diminution voire arrêt de l'immunosuppression, ce qui n'est pas toujours possible vu la nécessité des traitements immunosuppresseurs chez des patients atteints de maladies chroniques gravissimes, notamment les aplasies médullaires. D'autant plus ceci entraînerait un rebond de la maladie initiale. Ainsi, un traitement médicamenteux est souvent indiqué en association. Si les lésions sont agressives et nombreuses, on peut avoir recours à une électrocoagulation, une cryochirurgie au protoxyde d'azote ou au laser CO2 ou encore une monochimiothérapie par bléomycine ou par vincristine. En cas d'atteinte cutanée diffuse, muqueuse ou viscérale, le traitement systémique doit être envisagé, notamment une polychimiothérapie, une électrochiomiothérapie ou une radiothérapie [3].

Du fait de son action antinéoplasique et antiangiogénique, la rapamycine peut être proposée en remplacement d'un autre immunosuppresseur [12]. L'interféron alpha 2b recombinant est également proposé en intralésionnel ou intraveineux, mais ses effets indésirables et son coût limitent son utilisation [13]. Chez notre patient l'arrêt de l'immunosuppression seule était insuffisant. Un traitement par des cures de Bléomycine avait permis la rémission complète. La découverte d'HHV8 avait permis d'envisager de nouvelles perspectives thérapeutiques. Cependant, la place des antiviraux reste à ce jour mal déterminée [14].

## Conclusion

Peu d'auteurs ont rapportés la MK iatrogénique au cours d'une aplasie médullaire. Notre cas illustre l'intérêt d'un suivi régulier des patients sous immunosuppresseurs, notamment par un examen dermatologique systématique. Cette forme particulière de MK constitue un vrai défi thérapeutique pour le praticien.
